# Neuraminidase inhibitors during pregnancy and risk of adverse neonatal outcomes and congenital malformations: population based European register study

**DOI:** 10.1136/bmj.j629

**Published:** 2017-02-28

**Authors:** Sophie Graner, Tobias Svensson, Anna-Belle Beau, Christine Damase-Michel, Anders Engeland, Kari Furu, Anders Hviid, Siri Eldevik Håberg, Ditte Mølgaard-Nielsen, Björn Pasternak, Helle Kieler

**Affiliations:** 1 Department of Medicine, Centre for Pharmacoepidemiology, Karolinska Institutet, Karolinska University Hospital, SE-17176 Stockholm, Sweden; 2 Department of Obstetrics and Gynecology, Karolinska University Hospital, SE-17176 Stockholm, Sweden; 3 Service de Pharmacologie Médicale, CHU Toulouse, Université Toulouse III, UMR INSERM, FR-1027 Toulouse, France; 4 Division of Mental and Physical Health, Norwegian Institute of Public Health, P.O. Box 4404, Nydalen, NO-0403, Oslo, Norway; 5 Department of Global Public Health and Primary Care, University of Bergen, NO-5020 Bergen, Norway; 6 Department of Epidemiology Research, Statens Serum Institut, DK-2300, Copenhagen S, Denmark; 7 Clinical epidemiology Unit, Department of Medicine Solna, Karolinska Institutet, Karolinska University Hospital, SE-17176 Stockholm, Sweden

## Abstract

**Objective** To evaluate the possible effects of exposure to neuraminidase inhibitors during embryo-fetal life with respect to adverse neonatal outcomes and congenital malformations.

**Design** Population based multinational observational cohort study and meta-analysis.

**Setting** National registers covering information on maternal healthcare, births, and prescriptions in Denmark, Norway, and Sweden and the EFEMERIS database from the Haute-Garonne district in France.

**Participants** All women together with their singleton infants born between 1 January 2008 and 31 December 2010. Only infants born at 154 days of gestation or later were included. Infants were defined as exposed if the women filled a prescription during pregnancy for either of the two neuraminidase inhibitors oseltamivir or zanamivir.

**Main outcomes** Low birth weight, low Apgar score, preterm birth, small for gestational age birth, stillbirth, neonatal mortality, neonatal morbidity, and congenital malformations. Crude and adjusted hazard ratios of preterm birth were estimated using Cox regression models. Crude and adjusted odds ratios for other outcomes were estimated by logistic regression models.

**Results** The study included 5824 (0.8%) exposed women and their infants and 692 232 who were not exposed. Exposure to neuraminidase inhibitors in utero was not associated with increased risks of any of the investigated neonatal outcomes, including low birth weight (adjusted odds ratio 0.77, 95% confidence interval 0.65 to 0.91), low Apgar score (adjusted odds ratio 0.87, 0.67 to 1.14), preterm birth (adjusted hazard ratio 0.97, 0.86 to 1.10), small for gestational age birth (adjusted odds ratio 0.72, 0.59 to 0.87), stillbirth (adjusted odds ratio 0.81, 0.51 to 1.30), neonatal mortality (adjusted odds ratio 1.13, 0.56 to 2.28), and neonatal morbidity (adjusted odds ratio 0.92, 0.86 to 1.00). No increased risk of congenital malformations overall associated with maternal exposure was observed during the first trimester (adjusted odds ratio 1.06, 0.77 to 1.48). Similarly, no significantly increased risks of any of the outcomes were observed in an analysis restricted to oseltamivir alone.

**Conclusions** This large multinational register study found no increased risks of adverse neonatal outcomes or congenital malformations associated with exposure to neuraminidase inhibitors during embryo-fetal life. The results support previously reported findings that the use of neuraminidase inhibitors is not associated with increased risks of adverse fetal or neonatal outcomes.

## Introduction

Pregnant women are at increased risk of severe disease and death secondary to influenza infection. In addition, influenza and fever may increase the risk of adverse pregnancy outcomes.^1^ ^2^ ^3^ ^4^ Despite limited knowledge on the safety and effectiveness of neuraminidase inhibitors in pregnancy, the regulatory agencies in Europe and the US recommended treatment and post-exposure prophylaxis in patients at high risk, such as pregnant women, during the 2009-10 influenza A/H1N1 pandemic.^5^

In Europe, where our study took place, oseltamivir, taken orally, and zanamivir, which is inhaled, are the two approved neuraminidase inhibitors. Early treatment of influenza with oseltamivir has been associated with a reduced risk of severe infection and of admission to intensive care units for pregnant women.^1^ ^6^ Because of the 2009 H1N1 pandemic and the regulatory agencies’ recommendations, the number of women exposed to neuraminidase inhibitors during pregnancy increased markedly during the 2009-10 influenza pandemic compared with previous years.^7^ ^8^ Accordingly, in the next virulent seasonal influenza pandemic, a large number of women may be targeted for post-exposure prophylaxis and treatment globally.

No randomised controlled trials on use of neuraminidase inhibitors during pregnancy have been conducted. To date, seven observational studies including data from a total of approximately 2500 women who used oseltamivir during pregnancy found no increased risks of congenital malformations or other adverse pregnancy outcomes.^5^ ^9^ ^10^ ^11^ ^12^ ^13^ ^14^ The largest study so far was from Canada and included around 1200 exposed women. This study, which apparently had no information on timing of drug exposure, found a significantly lower risk (adjusted odds ratio 0.77) of having a moderately growth restricted infant (<10th centile) with oseltamivir.^10^ For congenital malformations, exposure during the first trimester is of relevance, and four of the other studies included exposure in the first trimester, collectively including approximately 300 women.^5^ ^12^ ^13^ ^14^ Their inadequate size and the fact that outcomes such as infant mortality, preterm births, severe intrauterine growth restriction, and congenital malformations occur rarely mean that the previous studies had limited statistical power for more detailed analyses of these outcomes.

Although the influenza pandemic in 2009-10 was relatively mild in most cases, the uncertainty about the aggressiveness of a next pandemic virus outbreak and the possibility of spreading in vulnerable populations, such as pregnant women, mean that preparedness is critical. This study was set up to assess risks of adverse birth outcomes in association with the two major and non-intravenously administered neuraminidase inhibitors, oseltamivir and zanamivir, when used during pregnancy. Its objective was to evaluate possible adverse effects of exposure to neuraminidase inhibitors in utero on outcomes of low birth weight, low Apgar score, preterm birth, small for gestational age birth, stillbirth, neonatal mortality, neonatal morbidity, and congenital malformations.

## Methods

We did a multinational population based register study, including all women and their singleton infants born between 1 January 2008 and 31 December 2010 in the Scandinavian countries (Denmark, Norway, and Sweden) and those recorded in the EFEMERIS database in France.^15^

### Setting and data sources

The Scandinavian countries have population based national registers, which include prospectively collected information for all inhabitants on demographic and health indicators such as births, dispensed drugs, and hospital contacts. The total population in the Scandinavian countries is approximately 20 million inhabitants, and the national parliaments have given informed consent to be included in the registers on behalf of their population.^16^ All registers include the civil registration number of each resident, a unique number assigned at birth or immigration, which allows linkage of individual data between registers. From the Scandinavian countries, we obtained data from the medical birth registers, the registers for prescribed drugs, and the patient registers. The Scandinavian medical birth registers include data on maternal characteristics, pregnancy, delivery, and the neonatal period, including information on maternal body mass index and on smoking during pregnancy. For congenital malformations, the data came from either the medical birth registers or the patient registers. All diagnoses and congenital malformations were recorded according to ICD-10 (international classification of diseases, 10th revision).^17^ The prescribed drug registers include data on dispensed substances, dispensed dose, package sizes, and formulations according to the Anatomical Therapeutic Chemical (ATC) classification of drugs together with the date of dispensing.^18^

From France, we included women recorded in the EFEMERIS database, which is described in detail elsewhere.^14^ ^15^ EFEMERIS is a population based database that holds health related data in the Haute-Garonne district, which has 1 million inhabitants. The register contains individual based information on prescribed drugs, pregnancy outcome, and neonatal health from national health insurance system, Mother and Child Protection Service, hospitals, and neonatal clinics. The data are anonymised before transmission to the pharmacology service.

From the various data sources, we constructed two separate databases: one including Scandinavian data (pooling data from the three countries into one database) and one with French data. Figure 1 describes the flow of data into the analysis.

**Figure f1:**
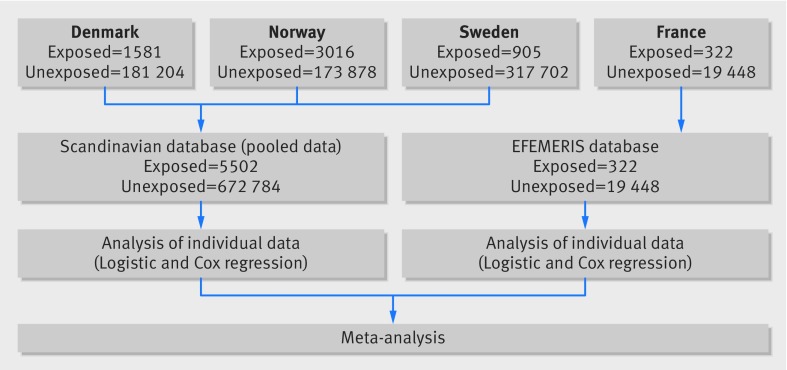
**Fig 1** Data flow in register based study on neuraminidase inhibitors during pregnancy

### Participants

All women together with their infants born during the study period and included in the Scandinavian birth registers or the EFEMERIS database were eligible for inclusion. Only singleton infants born at 154 days (22 weeks) of gestation or later were included. We excluded all infants (and their mothers) with an ICD-10 code suggesting chromosomal aberrations (Q90-99) or for whom the information on gestational age at delivery was missing. Women who had been admitted to hospital because of influenza or pneumonia (ICD-10 code J09-18) during pregnancy were excluded from the Scandinavian database. In the EFEMERIS database, information on hospital admission during pregnancy was not available so women admitted with influenza were not excluded in the French data.

### Patient involvement

No patients were involved in setting the research question or the outcome measures, nor were they involved in developing plans for design or implementation of the study. No patients were asked to advise on interpretation or writing up of results. There are no plans to disseminate the results of the research to study participants or the relevant patient community.

### Exposure to neuraminidase inhibitors during embryo-fetal life

We defined exposure as having filled a prescription after the date of assumed conception (that is, from 14 days after the first day of the last menstrual period), for either of the two neuraminidase inhibitors—oseltamivir (ATC code J05AH02) or zanamivir (ATC code JO5AH01)—as recorded in the prescribed drug registers or the EFEMERIS database. Last menstrual period was based on gestational age calculated at an obstetric ultrasound examination that most of the included women had in the first or second trimester. We defined first trimester as gestational day 0-90, second trimester as gestational day 91-180, and third trimester as gestational day 181 to delivery. For congenital malformations, we evaluated only exposures during the first trimester in the study. We analysed oseltamivir and zanamivir jointly in the study and assessed associations with exposure only to oseltamivir in sub-analyses. Potential confounders included infant’s country of birth (Scandinavian countries), infant’s year of birth, maternal age, maternal comorbidity, and smoking. We defined maternal comorbidity as having filled at least one prescription for drugs used to treat chronic diseases, such as hypertension or diabetes, within 365 days before the first day of the last menstrual period (Scandinavian data). For the French data, the corresponding period was three months before the last menstrual period. A complete list of the included ATC codes used as proxies for maternal disease is shown in supplementary table A.

### Outcome measures

We assessed the following neonatal outcomes: low birth weight, low Apgar score, preterm birth, small for gestational age birth, stillbirth, neonatal mortality, neonatal morbidity, and congenital malformations. We defined low birth weight as weighing less than 2500 g at birth regardless of gestational age, low Apgar as having an Apgar score of 0-6 at 5 minutes of life, preterm birth as birth before 259 gestational days (37 weeks), and small for gestational age birth as weighing 2 standard deviations or less below the corresponding mean birth weight by gestational length and infant sex in Sweden (Scandinavian data) or France (French data).^19^ Stillbirth was defined as recorded in the respective national registers or EFEMERIS database, neonatal mortality as death of a liveborn infant within the first 28 days of life, and neonatal morbidity as having one or more recorded ICD-10 codes P00-P99 within the first 28 days of life, grouped according to the affected organ system (supplementary table B). We defined malformations diagnosed within the first 28 days of life according to ICD-10 codes and the EUROCAT classification system.^20^ We did not consider minor malformations according to the EUROCAT classification.^20^

### Statistical analysis

The data in each database (Scandinavian countries and France) included information at the individual level, and in a first step we analysed the two databases separately. The Scandinavian database thus encompassed individual data from all three countries. We used logistic regression to estimate crude and adjusted odds ratios together with 95% confidence intervals for low birth weight, low Apgar score, small for gestational age birth, stillbirth, neonatal mortality, and neonatal morbidity. We used exact logistic regression for sparse data—that is, when no cases were reported among the exposed population. We used Cox proportional hazards regression models to estimate the hazards ratios together with 95% confidence intervals for preterm birth, with the gestational length in days as the underlying time variable. For the analyses of preterm birth, we followed women from gestational week 22 until an outcome event or censoring at a gestational age of 259 days (37 weeks). We considered women to be exposed from the day in pregnancy when a neuraminidase inhibitor was dispensed and to remain as such until delivery.

We adjusted all outcomes for country (Scandinavian database only), year of birth, maternal age, maternal comorbidity, and smoking. We excluded women from the adjusted analysis if they had missing data on any of the five covariates. For malformations, we present the adjusted odds ratios only from analyses with more than five births among exposed infants with the specific malformation or organ system event.

To assess the robustness of the logistic regression model and to investigate whether including body mass index in the model affected the results, we did a restricted analysis including only data from Sweden and Denmark, both of which had nearly complete information on this variable. As inclusion of body mass index in the analysis carried out in the subset did not confound the results, we did not consider this variable in further analyses.

To combine the adjusted results from the Scandinavian and the French databases, we did a meta-analysis using the Metan command (version 9) in Stata (version 11.2).^21^ ^22^ We estimated a random effect model. We used the χ^2^ test for heterogeneity and the I^2^ statistic to assess the heterogeneity between the Scandinavian and French data.

## Results

We included a total of 5824 (0.8%) exposed infants and 692 232 unexposed infants in the study—5502 exposed and 672 784 unexposed infants from the Scandinavian countries, and 322 exposed and 19 448 unexposed infants from France (fig 1). Among the exposed infants, most of the women (4310; 74%) had received oseltamivir and 441 were dispensed a neuraminidase inhibitor more than once during their pregnancy. Among the exposed women, 1220 were exposed in the first trimester, 2408 in the second trimester, and 2196 in the third trimester. Table 1 describes the study participants in more detail. Most of the exposed infants were born in 2010. The exposed women were slightly older and had a higher proportion of maternal comorbidity but a lower body mass index than unexposed women.

**Table 1 tbl1:** Maternal and infant characteristics by exposure to neuraminidase inhibitors during pregnancy. Values are numbers (percentages)

	Scandinavian database		EFEMERIS database	
	Exposed (n=5502)	Unexposed (n=672 784)	Exposed (n=322)	Unexposed (n=19 448)
**No of infants exposed**				
Denmark	1581 (28.7)	181 204 (26.9)	-	-
Norway	3016 (54.8)	173 878 (25.8)	-	-
Sweden	905 (16.4)	317 702 (47.2)	-	-
**Sex of infant**				
Female	2685 (48.8)	326 785 (48.6)	149 (46.3)	9303 (47.8)
Male	2816 (51.2)	345 853 (51.4)	167 (51.9)	9744 (50.1)
Missing information	1 (0)	146 (0)	6 (1.9)	401 (2.1)
**Year of birth**				
2008	4 (0.1)	223 989 (33.3)	0 (0)	5308 (27.3)
2009	1489 (27.1)	223 033 (33.2)	67 (20.8)	2794 (14.4)
2010	4009 (72.9)	225 762 (33.6)	255 (79.2)	11 346 (58.3)
**Mother’s age at delivery**				
≤19 years	98 (1.8)	9665 (1.4)	9 (2.8)	306 (1.6)
20-29 years	1943 (35.3)	273 477 (40.6)	136 (42.2)	8442 (43.4)
≥30 years	3461 (62.9)	389 597 (57.9)	177 (55.0)	10 700 (55.0)
Missing information	0	45 (0)	0	0
**Smoking during pregnancy**				
Yes	560 (10.2)	63 155 (9.4)	21 (6.5)	958 (4.9)
No	4464 (81.1)	574 020 (85.3)	287 (89.1)	16 997 (87.4)
Missing information	478 (8.7)	35 609 (5.3)	14 (4.3)	1493 (7.7)
**Maternal body mass index**				
≤24	1518 (27.6)	283 713 (42.2)	-	-
25-29	640 (11.6)	127 720 (19.0)	-	-
≥30	340 (6.2)	62 373 (9.3)	-	-
Missing information	3004 (54.6)	198 978 (29.6)		
**Maternal comorbidity** ^*^				
Yes	1375 (25.0)	125 686 (18.7)	72 (22.4)	3505 (18.0)
No	4127 (75.0)	547 098 (81.3)	250 (77.6)	15 943 (82.0)

*Defined as filled prescription for any drug used for chronic disease (supplementary table A) in one year (Scandinavia) or three month (France) period before last menstrual period.

### Neonatal outcomes

In the entire study population, stillbirth occurred in 4.1 per 1000 births and the neonatal mortality was 1.5 per 1000 live births. Similarly, 38 866 (5.6%) were born preterm, 24 164 (3.5%) had a low birth weight, 17 539 (2.5%) were small for gestational age, and 8503 (1.2%) had a low Apgar score.

Exposure to neuraminidase inhibitors in utero was not associated with increased risks of any of the investigated neonatal outcomes (table 2 and supplementary figure A). Exposure was associated with a decreased risk of being born at a low birth weight (adjusted odds ratio 0.77, 95% confidence interval 0.65 to 0.91) or small for gestational age (0.72, 0.59 to 0.87). We found no increased risk of being born preterm (adjusted hazard ratio 0.97, 0.86 to 1.10) or for stillbirth (adjusted odds ratio 0.81, 0.51 to 1.30).

**Table 2 tbl2:** Neuraminidase inhibitors during pregnancy and risks of neonatal outcomes (birth weight, Apgar score, preterm birth, small for gestational age birth, stillbirth, and neonatal mortality)

	Scandinavian database						EFEMERIS database				
	No (%)			Odds ratio (95% CI)			No (%)			Odds ratio (95% CI)	
	Exposed (n=5502)	Unexposed (n=672 784)		Unadjusted	Adjusted^*^		Exposed (n=322)	Unexposed (n=19 448)		Unadjusted	Adjusted^*^
**Birth weight**											
<2500 g	157 (2.9)	23 137 (3.4)		0.83 (0.70 to 0.97)	0.77 (0.65 to 0.91)		12 (3.7)	858 (4.4)		0.80 (0.45 to 1.44)	0.76 (0.42 to 1.41)
2500-4499 g	5151 (93.6)	624 953 (92.9)		Reference	Reference		293 (91.0)	16 768 (86.2)		Reference	Reference
≥4500 g	183 (3.3)	23 133 (3.4)		0.97 (0.83 to 1.12)	1.00 (0.86 to 1.17)		1 (0.3)	125 (0.6)		0.46 (0.06 to 3.32)	0.46 (0.06 to 3.31)
Missing information	11 (0.2)	1561 (0.2)		-	-		16 (5.0)	1697 (8.7)		-	-
**Apgar score**											
≤6	61 (1.1)	8345 (1.2)		0.89 (0.69 to 1.15)	0.87 (0.67 to 1.14)		0 (0)	97 (0.5)		0 (0 to 1.81)	0 (0 to 1.55)
≥7	5423 (98.6)	660 649 (98.2)		Reference	Reference		301 (93.5)	17 317 (89.0)		Reference	Reference
Missing information	18 (0.3)	3790 (0.6)		-	-		21 (6.5)	2034 (10.5)		-	-
**Preterm birth <37 weeks**											
Yes	268 (4.9)	37 145 (5.5)		1.00 (0.89 to 1.13)^†^	0.97 (0.86 to 1.11)^†^		20 (6.2)	1433 (7.4)		1.06 (0.68 to 1.65)^†^	0.97 (0.56 to 1.68)^†^
No	5234 (95.1)	635 639 (94.5)		Reference	Reference		302 (93.8)	18 015 (92.6)		Reference	Reference
**Small for gestational age** ^‡^											
Yes	111 (2.0)	17 046 (2.5)		0.80 (0.66 to 0.96)	0.72 (0.59 to 0.88)		4 (1.2)	378 (1.9)		0.61 (0.23 to 1.64)	0.60 (0.22 to 1.62)
No	5380 (97.8)	654 210 (97.2)		Reference	Reference		300 (93.2)	17 285 (88.9)		Reference	Reference
Missing information	11 (0.2)	1528 (0.2)		-	-		18 (5.6)	1785 (9.2)		-	-
**Stillbirth** ^§^											
Yes	14 (0.3)	2469 (0.4)		0.69 (0.41 to 1.17)	0.73 (0.41 to 1.29)		6 (1.9)	386 (2.0)		0.94 (0.42 to 2.12)	1.02 (0.45 to 2.31)
No	5488 (99.7)	670 315 (99.6)		Reference	Reference		315 (97.8)	19 013 (97.8)		Reference	Reference
Missing information	0 (0)	0 (0)		-	-		1 (0.3)	49 (0.3)		-	-
**Neonatal mortality**											
Yes	8 (0.1)	979 (0.1)		1.00 (0.50 to 2.00)	1.13 (0.56 to 2.28)		0 (0)	26 (0.1)		0 (0 to 7.39)	0 (0 to 67.49)
No	5480 (99.6)	669 336 (99.5)		Reference	Reference		315 (97.8)	18 987 (97.6)		Reference	Reference
Missing information	14 (0.3)	2469 (0.4)		-	-		7 (2.2)	435 (2.2)		-	-

*Adjusted for country (Scandinavian countries), year of birth, maternal age, maternal comorbidity. and smoking,

†Hazard ratio.

‡Corresponding to birth weight ≤2 standard deviations of national reference curve.

§EFEMERIS data not adjusted for smoking.

The analysis of neonatal morbidity included only Scandinavian data. We found no overall association between exposure to neuraminidase inhibitors and increased neonatal morbidity during the first 28 days of life (adjusted odds ratio 0.92, 0.86 to 1.00) (table 3). In the adjusted analysis, we found a decreased risk of respiratory and cardiovascular disorders specific to the neonatal period among infants exposed to neuraminidase inhibitors during pregnancy (adjusted odds ratio 0.84, 0.75 to 0.95). The estimates of risk for the neonatal outcomes by trimester of exposure and database are shown in supplementary table C.

**Table 3 tbl3:** Neuraminidase inhibitors during pregnancy and risks of neonatal morbidity by affected organ system in Scandinavian countries

Neonatal morbidity by organ system^*^	No (%)			Odds ratio (95% CI)	
	Exposed (n=5502)	Unexposed (n=672 784)		Unadjusted	Adjusted^†^
Fetus and newborn affected by maternal factors and by complications of pregnancy, labour, and delivery	117 (2.1)	10 940 (1.6)		1.32 (1.09 to 1.58)	0.94 (0.77 to 1.14)
Disorders related to length of gestation and fetal growth	293 (5.3)	35 632 (5.3)		1.01 (0.89 to 1.13)	0.89 (0.79 to 1.01)
Birth trauma	30 (0.5)	3248 (0.5)		1.13 (0.79 to 1.62)	0.85 (0.58 to 1.24)
Respiratory and cardiovascular disorders specific to perinatal period	314 (5.7)	34 630 (5.1)		1.12 (1.00 to 1.25)	0.84 (0.75 to 0.95)
Infections specific to perinatal period	68 (1.2)	8970 (1.3)		0.93 (0.73 to 1.18)	0.78 (0.60 to 1.00)
Haemorrhagic and haematological disorders of fetus and newborn	186 (3.4)	24 450 (3.6)		0.93 (0.80 to 1.07)	0.89 (0.77 to 1.04)
Transitory endocrine and metabolic disorders specific to fetus and newborn	114 (2.1)	13 541 (2.0)		1.03 (0.86 to 1.24)	0.84 (0.70 to 1.02)
Digestive system disorders of fetus and newborn	3 (0.1)	505 (0.1)		0.73 (0.23 to 2.27)	0.87 (0.28 to 2.74)
Conditions involving integument and temperature regulation of fetus and newborn	27 (0.5)	1961 (0.3)		1.69 (1.15 to 2.47)	1.07 (0.71 to 1.63)
Other disorders originating in perinatal period	222 (4.0)	19 753 (2.9)		1.39 (1.22 to 1.59)	0.98 (0.86 to 1.13)
All	912 (16.6)	96 773 (14.4)		1.18 (1.10 to 1.27)	0.92 (0.86 to 1.00)

*Numbers are given by diagnosis. ICD-10 codes (P00-P99) for morbidity per organ system are shown in supplementary table B.

†Adjusted for country, year of birth, maternal age, maternal comorbidity, and smoking.

When we included women exposed to only oseltamivir (supplementary table D), the point estimates and 95% confidence intervals for all the estimated outcomes were similar to those derived from the compound exposure (supplementary tables E-H and supplementary figure B).

### Congenital malformations

The analysis of congenital malformations included only Scandinavian data from infants exposed during the first trimester, as no congenital malformations were reported in the 95 infants exposed during the first trimester in the EFEMERIS database. Of the 1125 infants exposed in the first trimester, 44 had at least one congenital malformation diagnosed, yielding a prevalence of 3.9%. In the unexposed group, 19 509 (2.9%) of the 672 784 infants had at least one congenital malformation. Among the exposed infants, three (0.3%) had more than one malformation. The corresponding number for the unexposed infants was 2336 (0.3%). In the adjusted analysis, we found no increased risk of congenital malformations overall in association with exposure in utero (adjusted odds ratio 1.06, 0.77 to 1.48). Although the analysis was limited by small sample size, neuraminidase inhibitor exposure was not associated with increased risk of any of the specific subgroups of congenital malformations according to the EUROCAT classification. Table 4 shows the absolute numbers per organ system and the adjusted odds ratio together with 95% confidence intervals for malformations with more than five exposed births. The adjusted analysis of congenital malformations overall including infants exposed to oseltamivir in utero found an adjusted odds ratio of 1.08 (0.73 to 1.58).

**Table 4 tbl4:** Neuraminidase inhibitors during first trimester and congenital malformations in Scandinavian countries

Affected organ system^*^	No			Adjusted odds ratio (95% CI)^†^
	Exposed (n=1125)	Unexposed (n=672 784)	Total	
Nervous system	0	387	387	-
Eye	0	282	282	-
Ear, face, and neck	1	530	531	-
Heart	9	5360	5369	0.96 (0.48 to 1.93)
Respiratory	0	520	520	-
Cleft palate	3	840	843	-
Digestive organs	12	3118	3130	1.21 (0.62 to 2.33)
Abdominal organs	0	175	175	-
Urinary tract	7	2172	2179	1.31 (0.58 to 2.94)
Limbs	11	6768	6779	0.93 (0.50 to 1.73)
Skeleton	0	101	101	-
Skin	2	480	482	-
Greater arteries	2	1466	1468	-

*Numbers are given by diagnosis. Three exposed infants had malformations in more than one organ system; corresponding number among unexposed infants was 2336.

†Adjusted for country, year of birth, maternal age, maternal comorbidity, and smoking. Adjusted odds ratios with 95% CI are given for outcomes with more than five exposed infants.

## Discussion

In this large multinational population based study including almost 6000 infants exposed to neuraminidase inhibitors in utero, we found no significantly increased risks of adverse neonatal outcomes or congenital malformations. On the contrary, we observed a small decrease in risk of weighing less than 2500 g or being classified as small for gestational age after exposure to neuraminidase inhibitors. The decreased risk remained after adjustment for possible confounders such as infant’s year and country of birth and maternal age, comorbidity, and smoking during pregnancy. The findings of no increased risks remained when we restricted the analyses to exposure to oseltamivir only.

### Comparison with previous studies

The effect of neuraminidase inhibitors during pregnancy on neonatal outcomes and congenital malformations has been assessed previously.^2^ ^5^ ^9^ ^10^ ^11^ ^12^ ^13^ ^14^ ^23^ ^24^ However, this study is by far the largest, being almost twice the size of all other studies combined with respect to the number of exposed pregnant women, and our results confirm and expand on the findings in the previous studies of no association between neuraminidase inhibitors and a broad range of adverse neonatal outcomes. The previous studies, including a study from France with data overlapping ours,^14^ had limited statistical power to assess specific outcomes such as stillbirth,^5^ ^13^ ^14^ preterm birth,^2^ ^9^ ^10^ ^11^ ^13^ ^14^ ^24^ low Apgar score,^10^ ^11^ neonatal morbidity,^9^ ^13^ ^14^ mortality,^9^ ^13^ and congenital malformations.^5^ ^9^ ^12^ ^13^ ^14^

Previous studies that assessed the outcome of congenital malformations have collectively been based on approximately 300 women exposed in the first trimester and reported a total of 30 exposed cases of malformations.^5^ ^9^ ^12^ ^13^ ^14^ The 1125 women exposed in the first trimester and included in our study thus add substantially to the body of knowledge on possible adverse effects of neuraminidase inhibitors during pregnancy.

We observed an inverse association between exposure to neuraminidase inhibitors and the risk of low birth weight or intrauterine growth restriction, measured as infants born small for gestational age. A similar finding was reported in a study from Canada, including 1200 exposed women.^10^ In the Canadian study, a significant association with small for gestational age birth at less than 10th centile was seen (adjusted odds ratio 0.77, 0.60 to 0.98). Other than being a true association or a finding by chance, the protective effect of neuraminidase inhibitors on fetal growth may imply a “healthy user effect.” Women who filled a prescription for a neuraminidase inhibitor may have been under closer observation by their attending physician than their comparisons, which, at least in theory, could reduce the risk of growth restriction in the neonate. Despite adjusting the analyses for potential confounders related to health condition, such as age, smoking, and co-medication, we cannot preclude residual confounding.

In addition, a fetus may be exposed to several viruses with an adverse effect on fetal growth, and one may therefore speculate as to whether the reduced risk of small for gestational age birth could be partly due to the neuraminidase inhibitors affecting fetal growth in a positive direction through a general antiviral effect.^25^ Although oseltamivir (which constituted most of the exposure in the study) is converted to its active metabolite oseltamivir carboxylate, which may be transferred across the placenta, we find this explanation to be less likely.^26^ ^27^ ^28^ ^29^

### Strengths and limitations of study

Our study was population based and included data from four countries, which suggests good external validity of the results. The Scandinavian population based registers and the EFEMERIS database have previously provided robust data on exposure and outcomes.^30^ ^31^ The large study cohort with almost 6000 exposed women and their infants ensured sufficient statistical power to detect moderately increased risks of low birth weight, low Apgar score, preterm birth, growth restriction, and congenital malformations and to detect large increased risks of rare events such as stillbirth and neonatal mortality. However, with only 1125 women exposed in the first trimester, the study was not sufficiently powered to study specific congenital malformations. The information on exposure was collected prospectively, minimising the risk of recall bias. In contrast to several of the previous studies, we focused on the time period of the most recent influenza pandemic and included women and their infants born in 2008-10. Accordingly, we could ensure a high number of exposed infants in our study cohort, corresponding to almost 1% of the study population. An extended study period would have increased the number of exposed women only marginally, as use of neuraminidase inhibitors declined rapidly at the end of 2010.^7^ ^8^

We estimated exposure from the day when a prescription for a neuraminidase inhibitor was filled. The possibility exists that some of the women included in the study filled their prescription but did not take the drug or started taking it later than the date it was dispensed. This may lead to a possible bias by misclassification of exposure and a potential underestimation of the risks.

We did not have information on vaccination against H1N1 influenza among the women included in the study. All of the Scandinavian countries and France recommended vaccination for high risk groups, including pregnant women, during the pandemic. The vaccinations were free of charge and made accessible for all pregnant women, and the vaccination rate has been estimated to more than 50% among women in Sweden and Norway, although much lower in Denmark and France.^4^ ^32^ ^33^ ^34^ Influenza infection is associated with fetal death, and immunisation during pregnancy may have reduced the risk of influenza related fetal death during the pandemic.^4^ ^35^ Accordingly, our results of no association between exposure to neuraminidase inhibitors and fetal death may have been confounded by the vaccination status of the pregnant women, as exposure to neuraminidase inhibitors, presumably, was more prevalent among the non-vaccinated women.

In the studied countries, maternal healthcare is free of charge and widely accepted by the general population. Almost all pregnant women follow the national programmes, and post-exposure prophylaxis with neuraminidase inhibitors was recommended during the influenza epidemic of 2009-10 and was distributed free of charge or reimbursed to all pregnant women in Scandinavia and in France. In Norway (in contrast to the other participating countries) post-exposure prophylaxis with neuraminidase inhibitors was allowed to be dispensed by pharmacists to pregnant women without a prescription by a doctor. The dispensed drugs were, however, reported routinely to the Norwegian Prescription Database, and these women are included in our study. The internal validity of the study should be high, considering the absence of barriers to healthcare and access to post-exposure prophylaxis in the study population.

We excluded all women admitted to hospital because of influenza during pregnancy in the Scandinavian database. We did not have information on hospital admission during pregnancy for the French data. Hence, this much smaller cohort in the study may include sicker women than the larger Scandinavian cohort. However, the point estimates for the various outcomes were similar in both databases. The estimates in the meta-analysis show that the risk estimates changed only marginally when we combined the two sets of data. The rationale for the exclusion of women admitted to hospital in the Scandinavian data was to reduce confounding by indication, as we can assume that admitted women are sicker than women who receive outpatient care or post-exposure prophylaxis, as well as to safeguard the classification of exposure, as in-hospital drug use is not included in the prescribed drug registers at the individual level so there was no possibility to investigate whether admitted women had received neuraminidase inhibitors. In addition, we did not have information on the indication for the treatment—that is, whether it was prescribed as post-exposure prophylaxis or treatment for influenza. We assume that most the women were either taking the drug for a relatively non-severe illness or as post-exposure prophylaxis, as women admitted to hospital were excluded. As we excluded women admitted to hospital, our study population may have been healthier than those included in the previous studies.

This study was based on information from a Scandinavian database of almost 700 000 mother and infant pairs and the smaller French database EFEMERIS with almost 20 000 mother and infant pairs. In a previous study that was based on data from the national birth registers and the prescribed drug registers in the five Nordic countries—Scandinavia (Denmark, Norway, and Sweden), Finland, and Iceland—the results of analyses based on pooled data at the individual level were compared with those from an aggregate meta-analysis.^36^ The authors concluded that, for multi-database studies, the results from the two approaches to analysis would be similar. However, when data are sparse the pooled data are superior to a meta-analysis. We used both approaches in this study, optimising the information by pooling individual data from the Scandinavian countries and, as individual data from the French database could not be provided to the study analyst, doing an aggregate meta-analysis based on the results of the logistic and Cox regression analyses in the two databases.

We found no association between exposure to neuraminidase inhibitors and congenital malformations, although we did not study malformations in pregnancies ending before gestational week 22. If neuraminidase inhibitors cause severe malformations leading to a miscarriage or termination of pregnancy, our results of no increased risks of malformations may have been biased. However, in the French study, which included pregnancies ending before 22 weeks, no cases of termination of pregnancy due to malformation occurred among those exposed to oseltamivir.^14^

### Conclusion and clinical implications

In this large multinational register study, we found no increased risk of adverse neonatal outcomes or congenital malformations associated with exposure to neuraminidase inhibitors during pregnancy. Our results support previously reported findings that the use of neuraminidase inhibitors is not associated with increased risks of adverse fetal or neonatal outcomes.

**What is already known on this topic**

Seasonal influenza occurs yearly, and millions of pregnant women risk severe illness during seasons with a more aggressive strain
European and American health authorities have recommended treatment and post-exposure prophylaxis with neuraminidase inhibitors for pregnant women
Despite the recommendations, information on possible adverse effects on infants exposed to neuraminidase inhibitors during embryo-fetal life is limited

**What this study adds**

This is the largest study on the topic to date and includes almost 6000 infants exposed to neuraminidase inhibitors and around 700 000 unexposed infants
No increased risks of adverse neonatal outcomes including neonatal morbidity or mortality, poor fetal growth, low Apgar score, or congenital malformations were seen in exposed infants
The study supports previously reported findings that the use of neuraminidase inhibitors is not associated with increased risks of adverse fetal or neonatal outcomes

## Web Extra

Extra material supplied by the author

## Notes

Ethical approval: The study was granted ethical permission by the regional ethical committee in Stockholm (ref 2012/543-31/1).

Transparency statement: The lead author (the manuscript's guarantor) affirms that the manuscript is an honest, accurate, and transparent account of the study being reported; that no important aspects of the study have been omitted; and that any discrepancies from the study as planned have been explained.

Data sharing: No additional data available.
