# Supplementary material for: Neuraminidase inhibitors during pregnancy and risk of adverse neonatal outcomes and congenital malformations: population based European register study
Source: BMJ. 2017 Feb 28;356:j629. doi: 10.1136/bmj.j629 (PMC5421412; doi:10.1136/bmj.j629)
Supplement: Supplementary file 1 — Supplementary tables and figures [file gras035560.ww1.pdf]

Supplementary tables and figures

Supplementary table A: ATC codes used to define maternal co-morbidity.

Type of treatment	ATC code
Diabetic treatment	A10
Antithrombotic treatment	B01
Hypertensive treatment incl diuretics	C01-C03, C07-C09
Corticosteroids	H02
Antiviral treatment	J05 (except J05AH)
Cytotoxic and immuneregulation incl hormones	L01-L04
Neuroleptic and antipsychotic treatment incl sedatives	N05-N06
Asthma	R03

Supplementary table B: Neonatal morbidity by organ system (ICD-10 codes P00-P99).

Affected organ system	ICD 10
Fetus and newborn affected by maternal factors and by complications of pregnancy, labor and delivery	P00-P04
Disorders related to length of gestation and fetal growth	P05-P08
Birth trauma	P10-P15
Respiratory and cardiovascular disorders specific to the perinatal period	P20-P29
Infections specific to the perinatal period	P35-P39
Hemorrhagic and hematological disorders of fetus and newborn	P50-P61
Transitory endocrine and metabolic disorders specific to fetus and newborn	P70-P74
Digestive system disorders of fetus and newborn	P75-P78
Conditions involving the integument and temperature regulation of fetus and newborn	P80-P83
Other disorders originating in the perinatal period	P90-P96

Supplementary figure A: Neuraminidase inhibitors during pregnancy and risks of deviant birth weight, preterm birth, Small for Gestational Age and stillbirth from a random effect meta-analysis combining Scandinavian and French data.

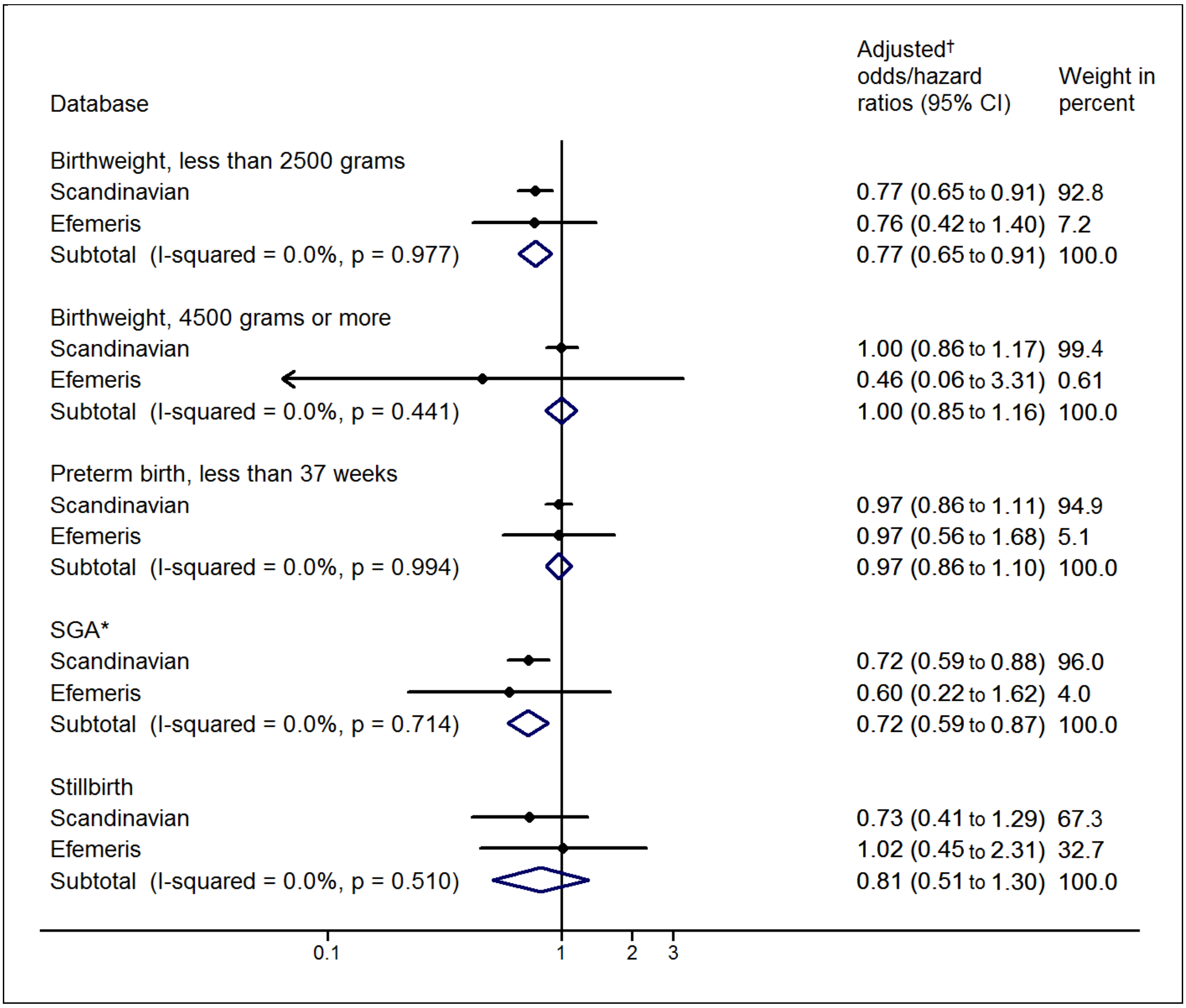

* Small for Gestational Age, corresponding to a birth weight ≤2 standard deviations of the national reference curve
‡Adjusted for country (only the Scandinavian database), year of birth, maternal age, maternal co-morbidity and smoking. Preterm birth presented as hazard ratio. Stillbirth not adjusted for smoking in EFEMERIS data.

Supplementary table C: Neuraminidase inhibitors during pregnancy and risks of neonatal outcomes (birth weight, Apgar score, preterm birth, Small for Gestational Age, stillbirth and neonatal mortality). Adjusted for country (Scandinavian countries), year of birth, maternal age, maternal co-morbidity and smoking. The results are presented per trimester of exposure and database.

	Scandinavian database								
	Exposed 1st trimester			Exposed 2nd trimester			Exposed 3rd trimetser		
	No (%)	Odds ratio (95% CI)		No (%)	Odds ratio (95% CI)		No (%)	Odds ratio (95% CI)	
	N=1125	Unadjusted	Adjusted ‡	N=2286	Unadjusted	Adjusted ‡	N=2091	Unadjusted	Adjusted ‡
**Birth weight (grams)**									
< 2500	41 (3.6)	1.06 (0.78 to 1.45)	0.95 (0.67 to 1.33)	73 (3.1)	0.93 (0.73 to 1.17)	0.88 (0.69 to 1.13)	43 (2.1)	0.59 (0.44 to 0.80)	0.56 (0.40 to 0.77)
2500-4499	1045 (92.9)	Ref	Ref	2127 (93.0)	Ref	Ref	1979 (94.6)	Ref	Ref
≥ 4500	38 (3.4)	0.98 (0.71 to 1.36)	1.04 (0.75 to 1.46)	80 (3.6)	1.02 (0.81 to 1.27)	1.07 (0.84 to 1.34)	65 (3.1)	0.9 (0.70 to 1.15)	0.91 (0.71 to 1.18)
Missing info	1 (0.1)			6 (0.3)			4 (0.2)		
**Apgar score**									
-6	18 (1.6)	1.29 (0.81 to 2.06)	1.17 (0.71 to 1.92)	25 (1.1)	0.88 (0.59 to 1.30)	0.90 (0.60 to 1.36)	18 (0.9)	0.69 (0.43 to 1.10)	0.67 (0.41 to 1.10)
7-	1104 (98.1)	Ref	Ref	2253 (98.6)	Ref	Ref	2066 (98.8)	Ref	Ref
Missing info	3 (0.3)			8 (0.3)			7 (0.3)		
**Preterm birth < 37 weeks[i]**									
Yes	65 (5.8)	1.06 (0.83 to 1.35)	1.03 (0.80 to 1.33)	131 (5.7)	1.04 (0.88 to 1.24)	0.99 (0.82 to 1.19)	72 (3.4)	0.90 (0.71 to 1.14)	0.90 (0.71 to 1.16)
No	1060 (94.2)	Ref	Ref	2155 (94.3)	Ref	Ref	2019 (96.6)	Ref	Ref
**SGA†**									
Yes	25 (2.2)	0.88 (0.59 to 1.30)	0.77 (0.50 to 1.19)	48 (2.1)	0.83 (0.62 to 1.10)	0.72 (0.53 to 0.98)	38 (1.8)	0.71 (0.52 to 0.98)	0.70 (0.50 to 0.97)
No	1099 (97.7)	Ref	Ref	2232 (97.6)	Ref	Ref	2049 (8.0)	Ref	Ref
Missing info	1 (0.1)			6 (0.3)			4 (0.2)		
**Stillbirth**									
Yes	3 (0.3)	0.73 (0.23 to 2.26)	0.90 (0.29 to 2.81)	5 (0.2)	0.60 (0.25 to 1.43)	0.73 (0.30 to 1.75)	6 (0.3)	0.78 (0.35 to 1.74)	0.64 (0.24 to 1.71)
No	1122 (99.7)	Ref	Ref	2281 (99.8)	Ref	Ref	2085 (99.7)	Ref	Ref
**Neonatal mortality**									
Yes	1 (0.1)	0.61 (0.09 to 4.34)	0.73 (0.10 to 5.19)	5 (0.2)	1.50 (0.62 to 3.62)	1.72 (0.71 to 4.18)	2 (0.1)	0.66 (0.16 to 2.63)	0.71 (0.18 to 2.87)
No	1121 (99.6)	Ref	Ref	2276 (99.6)	Ref	Ref	2083 (99.6)	Ref	Ref
Missing info	3 (0.3)			5 (0.2)			6 (0.3)		

	EFEMERIS database								
	Exposed 1st trimester			Exposed 2nd trimester			Exposed 3rd trimetser		
	**No (%)**	**Odds ratio (95% CI)**		**No (%)**	**Odds ratio (95% CI)**		**No(%)**	**Odds ratio (95% CI)**	
	N=95	**Unadjusted**	**Adjusted ‡**	N=122	**Unadjusted**	**Adjusted ‡**	N=105	**Unadjusted**	**Adjusted ‡**
**Birth weight (grams)**									
< 2500	4 (4.2)	1.92 (0.34 to 2.50)	1.01 (0.37 to 2.76)	7 (5.7)	1.30 (0.60 to 2.80)	1.17 (0.51 to 2.68)	1 (1.0)	0.19 (0.03 to1.39)	0.19 (0.02 to 1.36)
2500-4499	86 (90.5)	Ref	Ref	106 (86.9)	Ref	Ref	101 (96.0)	Ref	Ref
≥ 4500	0 (0.0)	0 (0 to 4.83)	0 (0 to 4.69)	0 (0.0)	0 (to 3.83)	0 (0 to 3.75)	1 (1.0)	1.38 (0.03 to 8.02)	1.55 (0.04 to 9.34)
Missing info	5 (5.3)			9 (7.4)			2 (2.0)		
**Apgar score**									
-6	0 (0.0)	0 (0 to 6.21)	0 (0 to 6.11)	0 (0.0)	0 (0 to 5.05)	0 (0 to 4.33)	0 (0.0)	0 (0 to 5.35)	0 (0 to 4.27)
7-	89 (93.4)	Ref	Ref	109 (89.3)	Ref	Ref	103 (98.0)	Ref	Ref
Missing info	6 (6.3)			13 (10.7)			2 (2.0)		
**Preterm birth < 37 weeks[i]**									
Yes	8 (8.4)	1.06 (0.48 to 2.36)	0.83 (0.27 to 2.60)	11 (9.0)	1.38 (0.78 to 2.43)	1.42 (0.70 to 2.85)	1 (1.0)	0.45 (0.11 to 1.79)	0.49 (0.12 to 1.95)
No	87 (91.6)	Ref	Ref	111 (91.0)	Ref	Ref	104 (99.0)	Ref	Ref
**SGA†**									
Yes	2 (2.1)	1.04 (0.26 to 4.24)	1.04 (0.25 to 4.24)	1 (0.9)	0.42 (0.06 to 2.99)	0.38 (0.05 to 2.75)	1 (1.0)	0.45 (0.06 to 3.22)	0.47 (0.06 to 3.37)
No	88 (92.6)	Ref	Ref	110 (90.1)	Ref	Ref	102 (97.0)	Ref	Ref
Missing info	5 (5.3)			11 (9.0)			2 (2.0)		
**Stillbirth ***									
Yes	3 (3.2)	1.61 (0.51 to 5.10)	1.63 (0.51 to 5.23)	3 (2.5)	1.25 (0.40 to 4.96)	1.30 (0.41 to 4.13)	0 (0.0)	0 (0 to 1.43)	0 (0 to 1.66)
No	92 (96.8)	Ref	Ref	118 (97.5)	Ref	Ref	105 (100)	Ref	Ref
Missing info	0 (0.0)			1 (0.0)			0 (0.0)		
**Neonatal mortality**									
Yes	0(0.0)	0 (0 to 25.58)	0 (0 to ω)	0 (0.0)	0 (0 to 19.88)	0 (0 to ω)	0 (0.0)	0 (0 to 22.38)	0 (0 to ω)
No	92 (96.8)	Ref	Ref	118 (96.6)	Ref	Ref	105 (100)	Ref	Ref
Missing info	3 (3.2)			4 (3.4)			0 (0.0)		

‡ Adjusted for country (Scandinavian countries), year of birth, maternal age, maternal co-morbidity and smoking, † Small for Gestational Age, corresponding to a birth weight ≤2 standard deviations of the national reference curve, [i] Presented as Hazard ratios, * Not adjusted for smoking in the EFEMERIS database

Supplementary table D: Maternal and infant characteristics by exposure to oseltamivir during pregnancy.

	Scandinavian database No (%)		EFEMERIS database No (%)	
	**Exposed** N=3989	**Unexposed** N=672784	**Exposed** N=321	**Unexposed** N=19448
**Number of infants exposed**				
Scandinavian country				
Denmark	1580 (39.6)	181204 (26.9)	-	-
Norway	1524 (38.2)	173878 (25.8)	-	-
Sweden	885(22.2)	317702 (47.2)	-	-
**Sex of the infant**				
Girl	1941 (48.7)	326785 (48.6)	149 (46.4)	9303 (47.8)
Boy	2047 (51.3)	345853 (51.4)	166 (51.7)	9744 (50.1)
Missing	1 (0.0)	146 (0.0)	6 (1.9)	401 (2.1)
**Year of birth**				
2008	4 (0.1)	223989 (33.3)	0 (0.0)	5308 (27.3)
2009	1131 (28.4)	223033 (33.2)	67 (20.9)	2794 (14.4)
2010	2854 (71.5)	225762 (33.6)	254 (79.1)	11346 (58.3)
**Mothers age at delivery (year)**				
- 19	64 (1.6)	9665 (1.4)	9 (2.8)	306 (1.6)
20 - 29	1394 (34.9)	273477 (40.6)	136 (42.4)	8442 (43.4)
30 -	2531 (63.4)	389597 (57.9)	176 (54.8)	10700 (55.0)
Missing information	0 (0.0)	45 (0.0)	0 (0.0)	0 (0.0)
**Smoking during pregnancy**				
Yes	393 (9.7)	63155 (9.4)	21 (6.5)	958 (4.9)
No	3334 (83.7)	574020 (85.3)	286 (89.1)	16997 (87.4)
Missing	262 (6.6)	35609 (5.3)	14 (4.4)	1493 (7.7)
**Maternal BMI**				
- 24	1454 (36.5)	283713 (4.3)	-	-
25 - 29	613 (15.4)	127720 (19.0)	-	-
30 -	332 (8.3)	62373 (9.3)	-	-
Missing information	1590 (39.8)	198978 (29.6)		
**Maternal co-morbidity ***				
Yes	1004 (25.2)	125686 (18.7)	72 (22.4)	3505 (18.0)
No	2985 (74.8)	547098 (81.3)	249 (77.6)	15943 (82.0)

*Defined as a filled a prescription for any of the medications used for chronic disease (supplementary table A) in the one year period prior before the last menstrual period (Scandinavia) or three months (France)

Supplementary table E: Oseltamivir during pregnancy and risks of neonatal outcomes (birth weight, Apgar score, preterm birth, Small for Gestational Age, stillbirth and neonatal mortality). Adjusted for country (Scandinavian countries), year of birth, maternal age, maternal co-morbidity and smoking.

	Scandinavian database				EFEMERIS database			
	No (%)		Odds ratio (95% CI)		No (%)		Odds ratio (95% CI)	
	Exposed N= 3989	Unexposed N=672784	Unadjusted	Adjusted ‡	Exposed N=321	Unexposed N= 19448	Unadjused	Adjusted ‡
**Birth weight (grams)**								
< 2500	121 (3.0)	23137 (3.4)	0.88 (0.73 to 1.05)	0.82 (0.68 to 1.00)	12 (3.7)	858 (4.4)	0.81 (0.45 to 1.44)	0.77 (0.42 to 1.41)
2500 to 4499	3715 (93.1)	624953 (93.0)	Ref	Ref	292 (91.0)	16768 (86.3)	Ref	Ref
≥ 4500	142 (3.6)	23133 (3.4)	1.04 (0.88 to 1.23)	1.09 (0.92 to 1.30)	1 (0.3)	125 (0.6)	0.46 (0.07 to 3.33)	0.46 (0.06 to 3.32)
Missing info	11 (0.3)	1561 (0.2)			16 (5.0)	1697 (8.7)		
**Apgar score**								
- 6	42 (1.0)	8345 (1.2)	0.85 (0.63 to 1.15)	0.87 (0.63 to 1.20)	0 (0.0)	97 (0.5)	0 (0 to 1.82)	0 (0 to 1.56)
7 -	3930 (98.5)	660649 (98.2)	Ref	Ref	300 (93.5)	17317 (89.0)	Ref	Ref
Missing info	17 (0.5)	3790 (0.6)			21 (6.5)	2034 (10.5)		
**Preterm birth < 37 weeks[i]**								
Yes	211 (5.3)	37145 (5.5)	1.10 (0.96 to 1.26)	1.05 (0.91 to 1.21)	20 (6.2)*	1433 (7.4)	1.07 (0.69 to 1.66)	0.98 (0.56 to 1.69)
No	3778 (94.7)	635639 (94.5)	Ref	Ref	301 (93.8)	18015 (92.6)	Ref	Ref
**SGA†**								
Yes	75 (1.9)	17046 (2.5)	0.74 (0.59 to 0.93)	0.66 (0.52 to 0.85)	4 (1.3)	378 (1.9)	0.61 (0.23 to 1.65)	0.60 (0.22 to 1.62)
No	3903 (97.8)	654210 (97.3)	Ref	Ref	299 (93.1)	17285 (88.9)	Ref	Ref
Missing info	11 (0.3)	1528 (0.2)			18 (5.6)	1785 (9.2)		
**Still birth***								
Yes	14 (0.4)	2469 (0.4)	0.96 (0.57 to 1.62)	0.98 (0.56 to 1.74)	6 (1.9)	386 (2.0)	0.94 (0.42 to 2.13)	1.02 (0.45 to 2.31)
No	3975 (99.6)	670315 (99.6)	Ref	Ref	314 (97.8)	19013 (97.8)	Ref	Ref
Missing info	0 (0.0)	0 (0.0)			1 (0.3)	49 (2.5)		
**Neonatal mortality**								
Yes	6 (0.2)	979 (0.1)	1.03 (0.46 to 2.31)	1.12 (0.50 to 2.51)	0 (0.0)	26 (0.2)	0 (0 to 7.42)	0 (0 to 67.49)
No	3969 (99.5)	669336 (99.5)	Ref	Ref	314 (97.8)	18987 (97.6)	Ref	Ref

Missing info	14 (0.3)	2469 (0.4)			7 (2.2)	435 (2.2)

‡Adjusted for country (Scandinavian countries), year of birth, maternal age, maternal co-morbidity and smoking,†Small for Gestational Age, corresponding to a birth weight ≤2 standard deviations of the national reference cure, [i] Presented as Hazard ratios, * not adjusted for smoking in EFEMERIS database

Supplementary table F: Oseltamivir during pregnancy and risks of neonatal morbidity by affected organ system in the Scandinavian countries.

	No (%)		Odds ratio(95% CI)	
	Exposed N=3989	Unexposed N= 672 784	**Unadjusted**	**Adjusted ‡**
**Neonatal morbidity by organ system***				
Fetus and newborn affected by maternal factors and by complications of pregnancy, labor and delivery	80 (2.0)	10 940 (1.6)	1.24 (0.99 to 1.55)	0.88 (0.70 to 1.11)
Disorders related to length of gestation and fetal growth	249 (6.2)	35 632 (5.3)	1.19 (1.05 to 1.35)	0.89 (0.78 to 1.02)
Birth trauma	22 (0.6)	3 248 (0.5)	1.14 (0.75 to 1.74)	0.85 (0.54 to 1.34)
Respiratory and cardiovascular disorders specific to the perinatal period	243 (6.1)	34 630 (5.1)	1.20 (1.05 to 1.36)	0.87 (0.75 to 0.99)
Infections specific to the perinatal period	56 (1.4)	8 970 (1.3)	1.06 (0.81 to 1.37)	0.92 (0.70 to 1.21)
Hemorrhagic and hematological disorders of fetus and newborn	165 (4.1)	24 450 (3.6)	1.14 (0.98 to 1.34)	0.98 (0.83 to 1.16)
Transitory endocrine and metabolic disorders specific to fetus and newborn	90 (2.3)	13 541 (2.0)	1.12 (0.91 to 1.39)	0.84 (0.67 to 1.04)
Digestive system disorders of fetus and newborn	3 (0.1)	505 (0.1)	1.00 (0.32 to 3.12)	1.03 (0.33 to 3.23)
Conditions involving the integument and temperature regulation of fetus and newborn	14 (0.4)	1 961 (0.3)	1.21(0.71 to 2.04)	0.92 (0.52 to 1.63)
Other disorders originating in the perinatal period	145 (3.6)	19 753 (2.9)	1.25 (1.06 to 1.47)	0.86 (0.72 to 1.02)
All	707 (17.7)	96 773 (14.4)	1.28 (1.18 to 1.39)	0.93 (0.85 to 1.01)

* Numbers are given by diagnosis ICD 10 codes (P00-P99) for morbidity per organ system are presented in supplementary table B, ‡ Adjusted for country , year of birth, maternal age, maternal co-morbidity and smoking

Supplementary table G: Oseltamivir during first trimester and congenital malformations in the Scandinavian countries. Adjusted for country, year of birth, maternal age, maternal co-morbidity and smoking.

	No			Odds ratio (95% CI)
**Affected organasystem** *	**Exposed** N=814	**Unexposed** N=672784	**Total**	**Adjusted ‡**
Nervous System	0	388	388	
Eye	0	282	282	
Ear, face and neck	1	533	534	
Heart	7	5391	5398	0.96 (0.43 to 2.15)
Respiratory	0	522	522	
Cleft palate	3	844	847	
Digestive organs	7	3137	3144	1.18 (0.53 to 2.65)
Abdominal organs	0	175	175	
Urinary tract	5	2179	2184	1.54 (0.64 to 3.75)
Limbs	7	6793	6800	0.88 (0.42 to 1.85)
Skeleton	0	101	101	
Skin	1	482	483	
Greater artieries	1	1472	1473	

* Numbers are given by diagnosis, and two of the exposed infants had malformations in more than one organ system. The corresponding number among the unexposed was 2336. ‡ Adjusted odds ratios with 95% confidence intervals are presented for outcomes with more than five exposed cases. Adjusted for country, year of birth, maternal age, maternal co-morbidity and smoking

Supplementary table H: Oseltamivir during pregnancy and risks of neonatal outcomes (birth weight, Apgar score, preterm birth, Small for Gestational Age, stillbirth and neonatal mortality). Adjusted for country (Scandinavian countries), year of birth, maternal age, maternal co-morbidity and smoking. The results are presented per trimester of exposure and database.

	**Scandinavian database**								
	**Exposed 1st trimester**			**Exposed 2nd trimetser**			**Exposed 3rd trimester**		
	**No (%)** N= 814	**Odds ratio (95% CI)**		**No (%)** N=1661	**Odds ratio (95% CI)**		**No (%)** N=1514	**Odds ratio (95% CI)**	
		**Undjusted**	**Adjusted**		**Undjusted**	**Adjusted**		**Undjusted**	**Adjusted**
**Birth weight (grams)**									
< 2500	31 (3.8)	1.11 (0.78 to 1.59)	0.95 (0.64 to 1.41)	58 (3.5)	1.01 (0.78 to 1.32)	1 (0.76 to 1.31)	32 (2.1)	0.61 (0.43 to 0.86)	0.56 (0.38 to 0.81)
2500 - 4499	754 (92.6)	Ref	Ref	1535 (92.4)	Ref	Ref	1426 (94.2)	Ref	Ref
≥ 4500	28 (3.4)	1 (0.69 to 1.46)	1.1 (0.75 to 1.61)	62 (3.7)	1.09 (0.85 to 1.41)	1.16 (0.90 to 1.51)	52 (3.4)	1.00 (0.76 to 1.32)	1 (0.75 to 1.33)
Missing info	1 (0.1)			6 (0.4)			4 (0.3)		
**Apgar score**									
- 6	15 (1.8)	1.49 (0.90 to 2.49)	1.36 (0.78 to 2.36)	17 (1.0)	0.82 (0.51 to 1.33)	0.95 (0.59 to 1.53)	10 (0.6)	0.53 (0.28 to 0.99)	0.5 (0.25 to 0.99)
7 -	796 (97.8)	Ref	Ref	1636 (98.5)	Ref	Ref	1498 (98.9)	Ref	Ref
Missing info	3 (0.4)			8 (0.5)			6 (0.5)		
**Preterm birth < 37 weeks[i]**									
Yes	54 (6.6)	1.22 (0.93 to 1.59)	1.16 (0.88 to 1.53)	100 (6.0)	1.1 (0.90 to 1.33)	1.03 (0.84 to 1.27)	57 (3.8)	1 .00 (0.76 to 1.30)	0.99 (0.75 to 1.30)
No	760 (93.4)	Ref	Ref	1561 (94.0)	Ref	Ref	1457 (96.2)	Ref	Ref
**SGA†**									
Yes	18 (2.2)	0.87 (0.55 to 1.39)	0.69 (0.41 to 1.17)	34 (2.0)	0.81 (0.57 to 1.13)	0.74 (0.52 to 1.06)	23 (1.5)	0.6 (0.40 to 0.90)	0.56 (0.36 to 0.86)
No	795 (97.7)	Ref	Ref	1621 (97.6)	Ref	Ref	1487 (98.2)	Ref	Ref
Missing info	1 (0.1)			6 (0.4)			4 (0.3)		
**Still birth**									

Yes	3 (0.4)	1.00 (0.32 to 3.12)	1.21 (0.39 to 3.78)	5 (0.3)	0.82 (0.34 to 1.97)	0.99 (0.41 to 2.38)	6 (0.4)	1.08 (0.48 to 2.41)	0.86 (0.32 to 2.30)
No	811 (99.6)	Ref	Ref	1656 (99.7)	Ref	Ref	1508 (99.6)	Ref	Ref
**Neonatal mortality**									
Yes	1 (0.1)	0.84 (0.12 to 6.01)	0.95 (0.13 to 6.79)	4 (0.2)	1.66 (0.62 to 4.43)	1.81 (0.67 to 4.86)	1 (0.1)	0.45 (0.06 to 3.23)	0.48 (0.07 to 3.40)
No	810 (99.5)	Ref	Ref	1652 (99.5)	Ref	Ref	1507 (99.5)	Ref	Ref
Missing info	3 (0.4)			5 (0.3)			6 (0.4)		

	EFEMERIS database								
	Exposed 1st trimester			Exposed 2nd trimetser			Exposed 3rd trimester		
	No (%) N= 94	Odds ratio (95% CI)		No (%) N=122	Odds ratio (95% CI)		No (%) N=105	Odds ratio (95% CI)	
		Undjusted	Adjusted		Undjusted	Adjusted		Undjusted	Adjusted
**Birthweight (grams)**									
< 2500	4 (4.3)	0.93 (0.34 to 2.53)	1.02 (0.37 to 2.79)	7(5.7)	1.3 (0.60 to 2.80)	1.17 (0.51 to 2.68)	1 (1.0)	0.19 (0.03 to 1.39)	0.19 (0.03 to 1.36)
2500 - 4499	85 (90.4)	Ref	Ref	106 (86.9)	Ref	Ref	101 (96.2)	Ref	Ref
≥ 4500	0 (0.0)	0 (0 to 4.88)	0 (0 to 4.74)	0 (0.0)	0 (0 to 3.83)	0 (0 to 3.75)	1 (1.0)	1.38 (0.03 to 8.02)	0 (0 to 9.34)
Missing info	5 (5.3)			9 (7.4)			2 (1.8)		
**Apgar score**									
- 6	0 (0.0)	0 (0 to 6.28)	0 (0 to 6.19)	0 (0.0)	0 (0 to 5.05)	0 (0 to 4.33)	0 (0.0)	0 (0 to 5.35)	0 (0 to 4.27)
7 -	88 (93.6)	Ref	Ref	109 (89.3)	Ref	Ref	103 (98.1)	Ref	Ref
Missing	6 (6.4)			13 (10.7)			2 (1.9)		
**Preterm birth < 37 weeks[i]**									
Yes	8 (8.5)	1.07 (0.48 to 2.40)	0.85 (0.27 to 2.63)	11 (9.0)	1.38 (0.78 to 2.43)	1.42 (0.70 to 2.85)	1 (1.0)	0.45 (0.11 to 1.79)	0.49(0.12 to 1.95)
No	86 (91.5)	Ref	Ref	111(91.0)	Ref	Ref	104 (99.0)	Ref	Ref
**SGA†**									
Yes	2 (2.2)	1.05 (0.26 to 4.29)	1.05 (0.26 to 4.29)	1 (0.8)	0.42 (0.06 to 2.99)	0.38 (0.05 to 2.75)	1 (1.0)	0.45 (0.06 to 3.22)	0.47 (0.06 to 3.37)
No	87 (93.5)	Ref	Ref	110 (90.2)	Ref	Ref	102 (97.1)	Ref	Ref
Missing info	5 (5.3)			11 (9.0)			2 (1.9)		
**Still birth ***									
Yes	3 (3.2)	1.62 (0.51 to 5.15)	1.65 (0.52 to 5.29)	3 (2.5)	1.25 (0.40 to 3.96)	1.30 (0.41 to 4.13)	0 (0.0)	0 (0 to 1.43)	0 (0 to 1.66)
No	91 (96.8)	Ref	Ref	118 (96.7)	Ref	Ref	105 (100)	Ref	Ref
Missing info	0 (0.0)			1 (0.8)			0 (0.0)		
**Neonatal mortality**									
Yes	0 (0.0)	0 (0 to 25.87)	0 (0 to ω)	0 (0.0)	0 (0 to 19.88)	0 (0 to ω)	0 (0.0)	0 (0 to 22.38)	0 (0 to ω)
No	91 (96.8)	Ref	Ref	118 (96.7)	Ref	Ref	105 (100)	Ref	Ref
Missing info	3 (3.2)			4 (3.3)			0 (0.0)		

‡ Adjusted for country (Scandinavian countries), year of birth, maternal age, maternal co-morbidity and smoking, † Small for Gestational Age, corresponding to a birth weight ≤2 standard deviations of the national reference curve, [i] Presented as Hazard ratios, * Not adjusted for smoking in the EFEMERIS database

Supplementary figure B: Oseltamivir during pregnancy and risks of deviant birth weight, preterm birth, Small for Gestational Age and stillbirth from a random effect meta-analysis combining Scandinavian and French data.

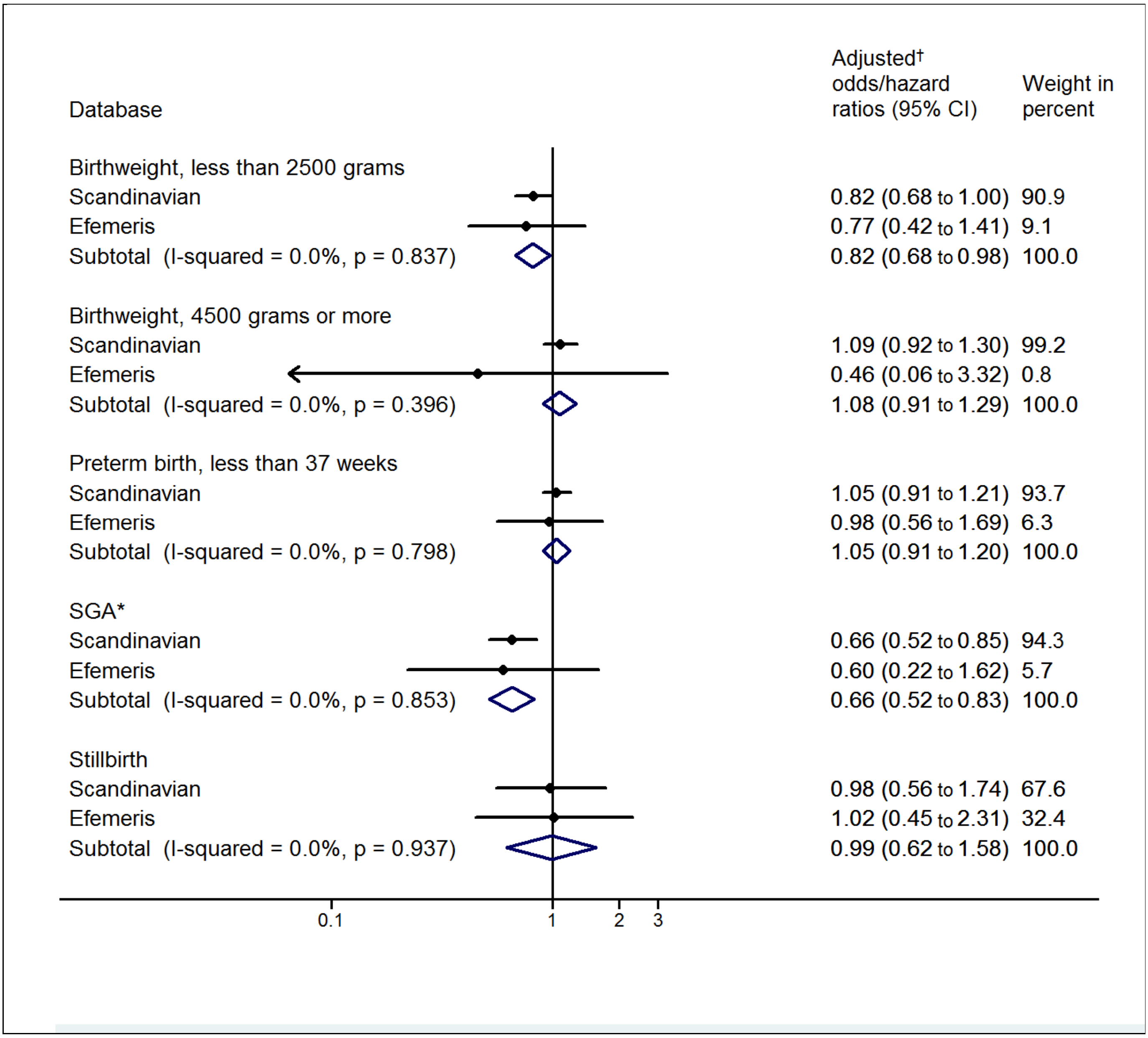

* Small for Gestational Age, corresponding to a birth weight ≤2 standard deviations of the national reference curve † Adjusted for country (only the Scandinavian database), year of birth, maternal age, maternal co-morbidity and smoking. Preterm birth presented as hazard ratio. Stillbirth not adjusted for smoking in EFEMERIS data.
